# Supplementary figures and images for: Pandemic trends in health care use: From the hospital bed to self-care with COVID-19
Source: PLoS One. 2022 Mar 23;17(3):e0265812. doi: 10.1371/journal.pone.0265812 (PMC8942224; doi:10.1371/journal.pone.0265812)

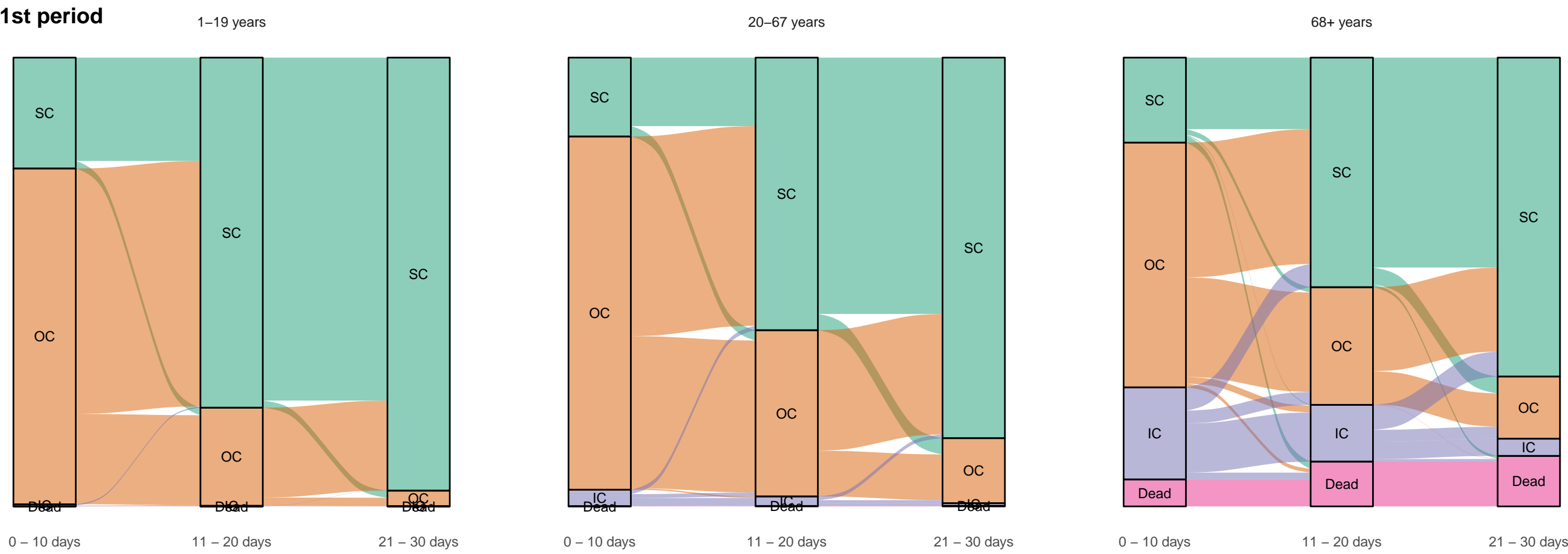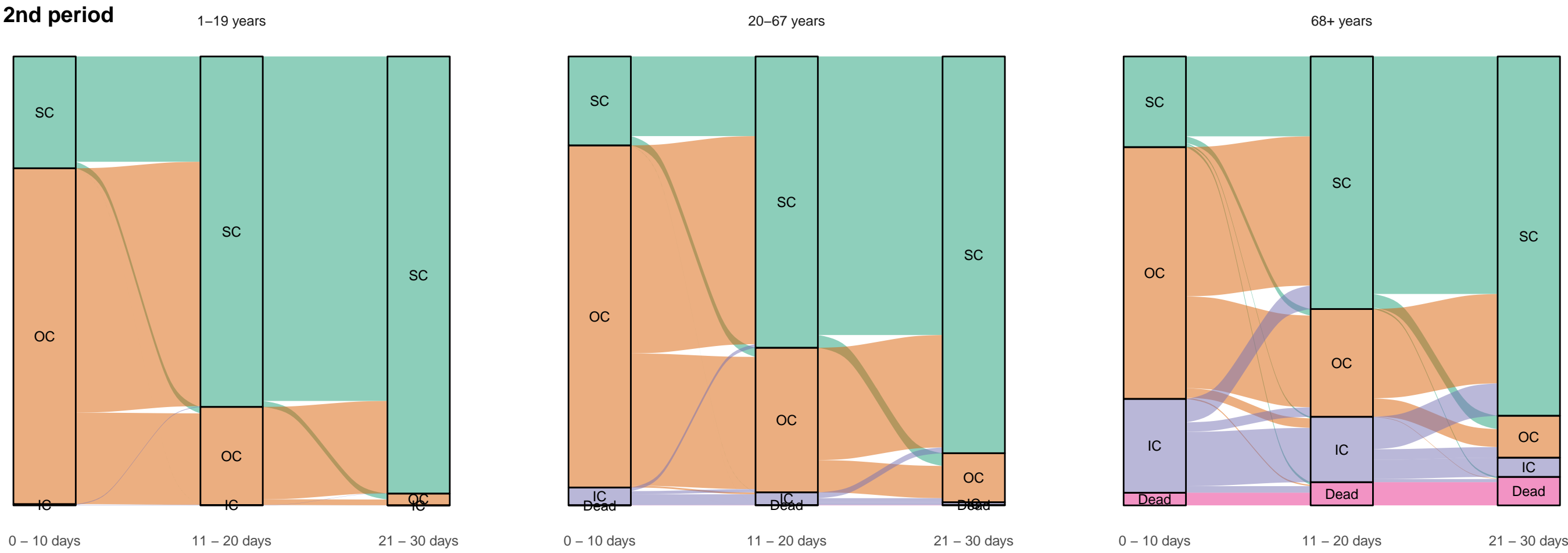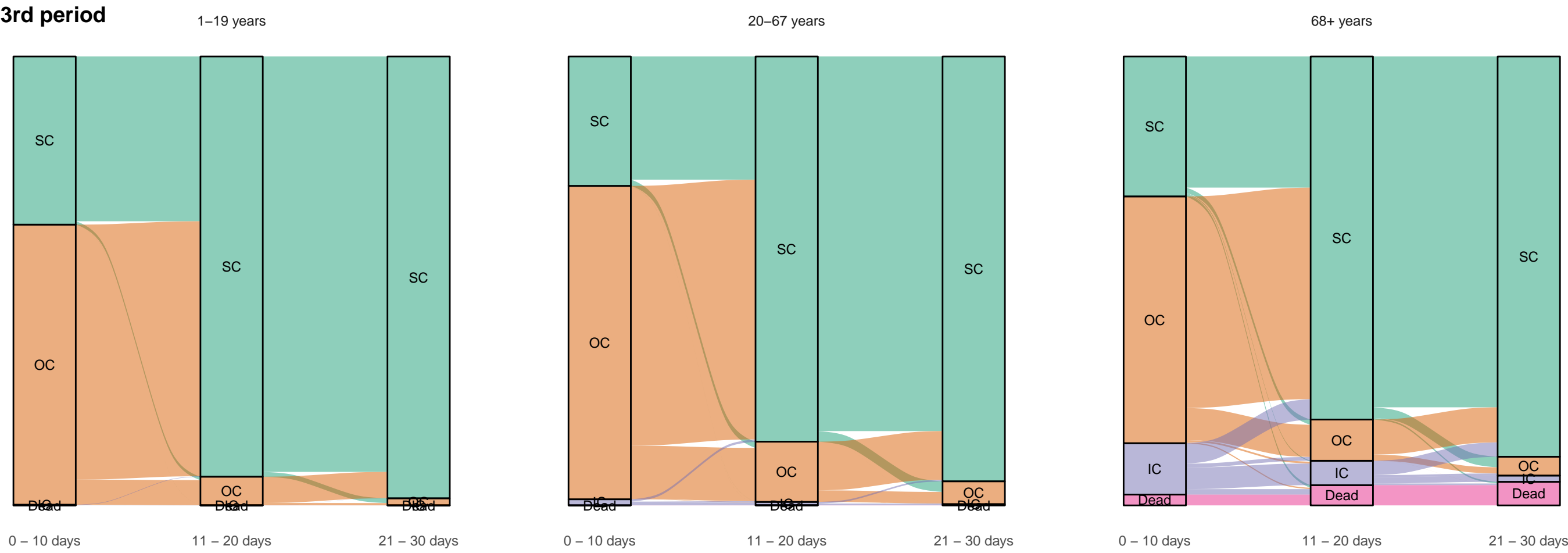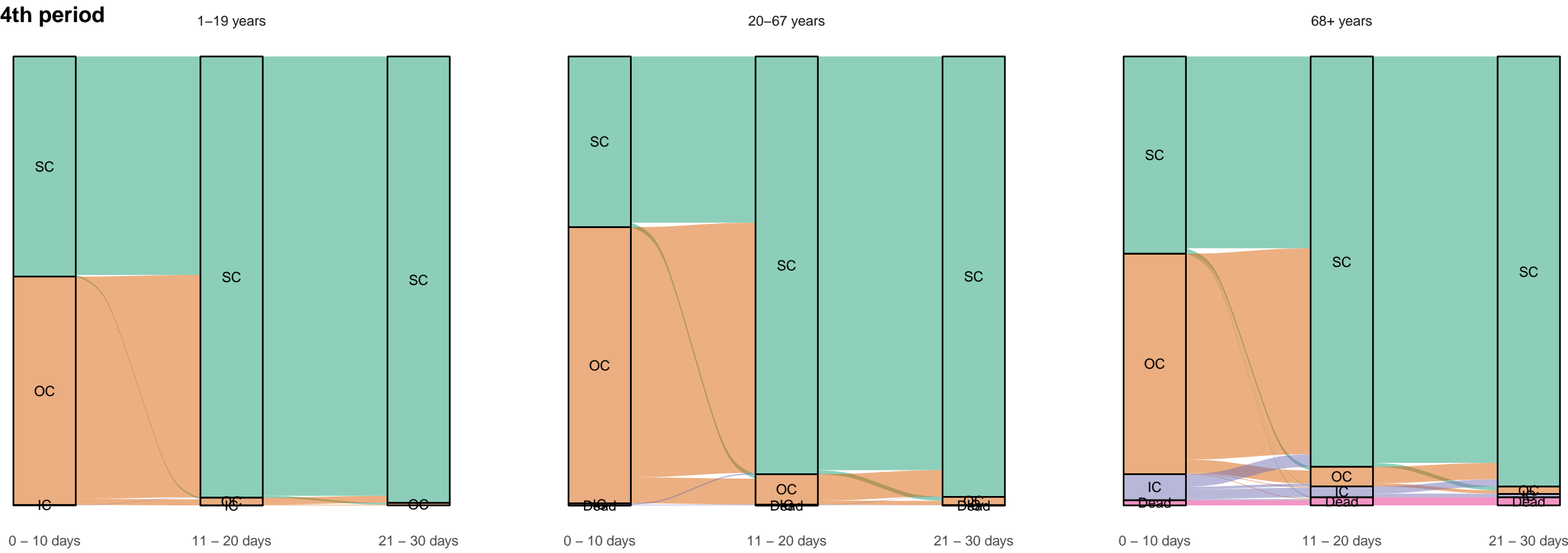

Supplement: S1 Fig — Note: This figure visualizes the most common pathway-flows for each period and each age-group. SC = Self-care; OC = Outpatient care; IC = Inpatient care; and Dead = Dead within 30 days. Colors show which strata each person belonged to in the last 10 days. Categories are mutually exclusive and ordered by severeness (Death > IC > OC > SC). Crude numbers are removed in order to avoid confusion. The sizes of each stratum should therefore be interpreted as shares. (PDF) [file pone.0265812.s001.pdf]

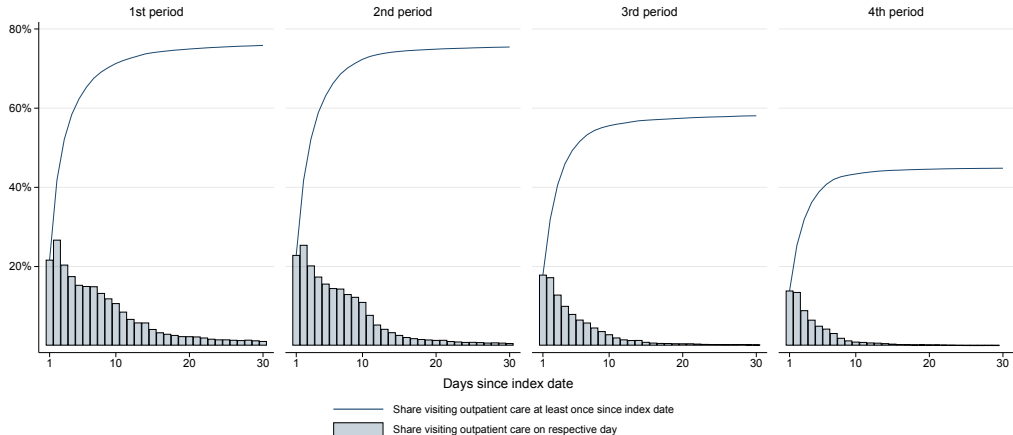

Supplement: S3 Fig — (PDF) [file pone.0265812.s003.pdf]

1st period

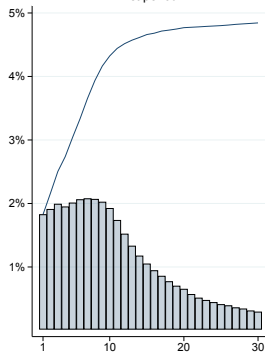

2nd period

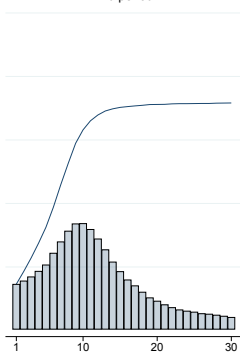

3rd period

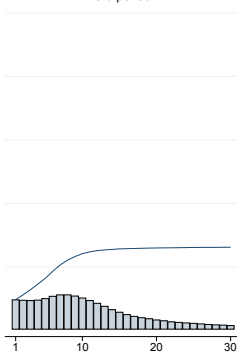

4th period

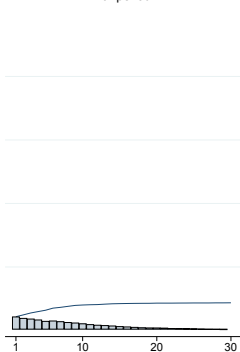

Days since index date

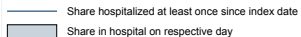

Supplement: S5 Fig — (PDF) [file pone.0265812.s005.pdf]
