# Supplementary material for: Pandemic trends in health care use: From the hospital bed to self-care with COVID-19
Source: PLoS One. 2022 Mar 23;17(3):e0265812. doi: 10.1371/journal.pone.0265812 (PMC8942224; doi:10.1371/journal.pone.0265812)
Supplement: S2 Fig — Note: Day 0 includes 0–2 days before testing positive. (PDF) [file pone.0265812.s002.pdf]

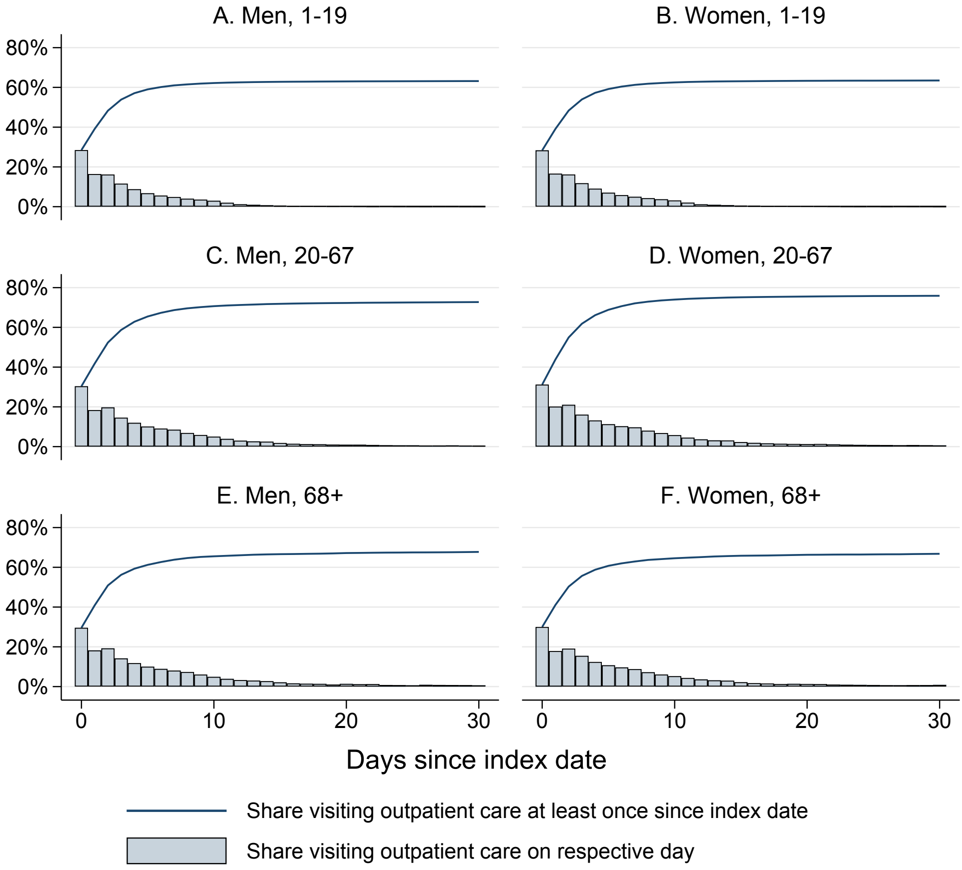
A. Men, 1-19
B. Women, 1-19
C. Men, 20-67
D. Women, 20-67
E. Men, 68+
F. Women, 68+
80%
60%
40%
20%
0%
0
10
20
30
Days since index date
Share visiting outpatient care at least once since index date
Share visiting outpatient care on respective day
