# Supplementary material for: Pandemic trends in health care use: From the hospital bed to self-care with COVID-19
Source: PLoS One. 2022 Mar 23;17(3):e0265812. doi: 10.1371/journal.pone.0265812 (PMC8942224; doi:10.1371/journal.pone.0265812)
Supplement: S4 Fig — Note: Day 0 includes 0–2 days before testing positive. (PDF) [file pone.0265812.s004.pdf]

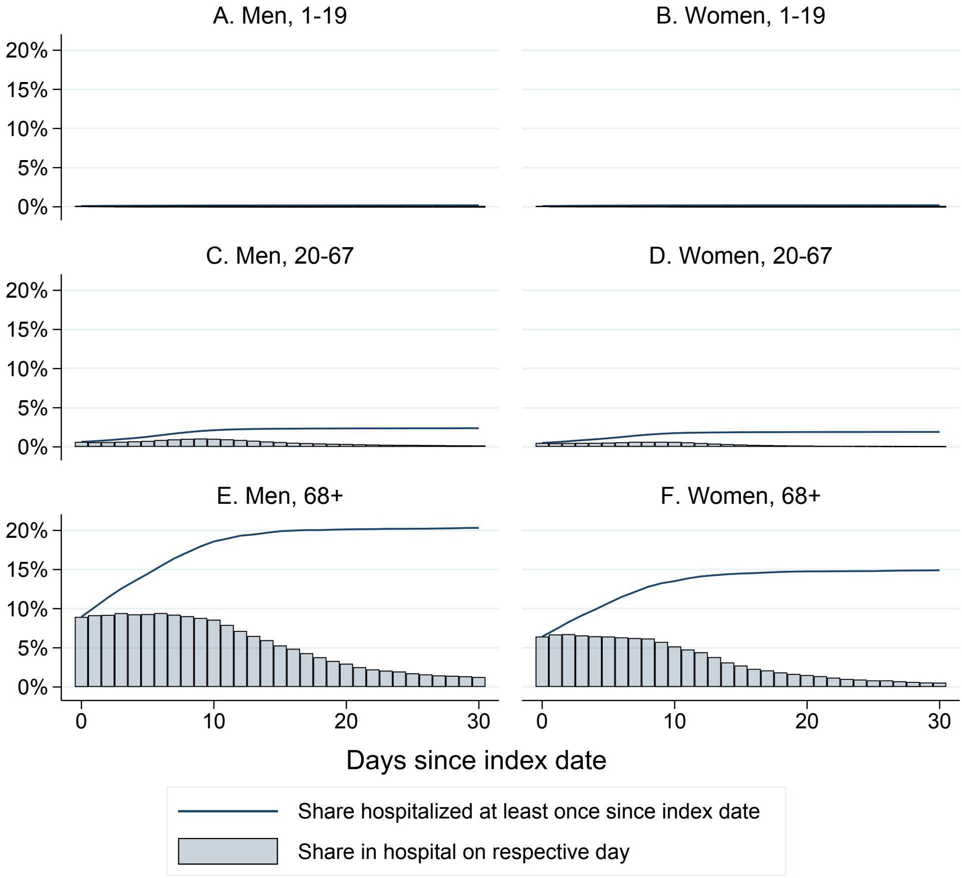
A. Men, 1-19
B. Women, 1-19
C. Men, 20-67
D. Women, 20-67
E. Men, 68+
F. Women, 68+
20%
15%
10%
5%
0%
0
10
20
30
Days since index date
Share hospitalized at least once since index date
Share in hospital on respective day
