# Supplementary material for: Pandemic trends in health care use: From the hospital bed to self-care with COVID-19
Source: PLoS One. 2022 Mar 23;17(3):e0265812. doi: 10.1371/journal.pone.0265812 (PMC8942224; doi:10.1371/journal.pone.0265812)
Supplement: S1 Table — Note: Due to privacy reasons we cannot report exact numbers when numbers are between 0 and 5. Therefore we have censured the exact numbers for deaths in the 1st and 3rd wave for the various age groups. The table still includes the percentages and 95% confidence intervals. (PDF) [file pone.0265812.s006.pdf]

S1 Table: Outcomes of persons testing positive for SARS-CoV-2 within 30 days, in each of four pandemic waves in Norway, 2020-2022.

		Self-care	Outpatient care	Inpatient care	All-cause mortality
Children and adolescents (1-19 years)					
1st wave		2,849 (22.5%)	9,775 (77.3%)	67 (0.5%)	- (0.0%)
	95% CI	*[21.8%-23.3%]*	*[76.6%-78.1%]*	*[0.4%-0.7%]*	*[0.0%-0.0%]*
2nd wave		5,003 (23.1%)	16,632 (76.8%)	65 (0.3%)	0
	95% CI	*[22.5%-23.7%]*	*[76.2%-77.4%]*	*[0.2%-0.4%]*	
3rd wave		37,180 (36.5%)	64,726 (63.5%)	149 (0.1%)	- (0.0%)
	95% CI	*[36.2%-36.8%]*	*[63.2%-63.8%]*	*[0.1%-0.2%]*	*[0.0%-0.0%]*
4th wave		21,132 (48.5%)	22,454 (51.5%)	43 (0.1%)	0
	95% CI	*[48.0%-48.9%]*	*[51.0%-52.0%]*	*[0.1%-0.1%]*	
Adults in working age (20-67 years)					
1st wave		6,399 (13.6%)	40,304 (85.8%)	1,928 (4.1%)	- (0.1%)
	95% CI	*[13.3%-13.9%]*	*[85.5%-86.1%]*	*[3.9%-4.3%]*	*[0.1%-0.2%]*
2nd wave		6,749 (16.5%)	33,931 (83.1%)	1,803 (4.4%)	38 (0.1%)
	95% CI	*[16.2%-16.9%]*	*[82.0%-82.7%]*	*[4.2%-4.6%]*	*[0.1%-0.1%]*
3rd wave		35,419 (26.6%)	97,432 (73.1%)	1,937 (1.5%)	- (0.1%)
	95% CI	*[26.3%-26.8%]*	*[72.9%-73.3%]*	*[1.4%-1.5%]*	*[0.0%-0.1%]*
4th wave		27,067 (36.3%)	47,342 (63.5%)	369 (0.5%)	8 (0.0%)
	95% CI	*[36.0%-36.7%]*	*[63.2%-63.8%]*	*[0.4%-0.5%]*	*[0.0%-0.0%]*
Elderly (68 years)					
1st wave		693 (15.0%)	3,470 (74.8%)	1,134 (24.5%)	- (11.3%)
	95% CI	*[14.0%-16.0%]*	*[73.6%-76.1%]*	*[23.3%-25.7%]*	*[10.4%-12.2%]*
2nd wave		287 (16.9%)	1,287 (76.0%)	426 (25.2%)	108 (6.3%)
	95% CI	*[15.2%-18.8%]*	*[73.9%-77.9%]*	*[23.1%-27.3%]*	*[5.3%-7.6%]*
3rd wave		2,643 (28.5%)	6,042 (65,1%)	1,243 (13.4%)	- (5.3%)
	95% CI	*[27.6%-29.4%]*	*[64.1%-66.1%]*	*[12.7%-14.1%]*	*[4.8%-5.7%]*
4th wave		1,017 (42.2%)	1,311 (54.4%)	158 (6.6%)	44 (1.8%)
	95% CI	*[40.2%-44.1%]*	*[52.4%-56.3%]*	*[5.6%-7.6%]*	*[1.3%-2.4%]*
Total (all age groups)					
1st wave		9,941 (15.5%)	53,549 (83.3%)	3,129 (4.9%)	590 (0.9%)
	95% CI	*[15.2%-15.8%]*	*[83.0%-83.6%]*	*[4.7%-5.0%]*	*[0.8%-1.0%]*
2nd wave		12,039 (18.8%)	51,850 (80,8%)	2,294 (3.6%)	146 (0.2%)
	95% CI	*[18.5%-19.1%]*	*[80.5%-81.1%]*	*[3.4%-3.7%]*	*[0.2%-0.3%]*
3rd wave		75,242 (30.8%)	168,200 (68,8%)	3,329 (1.4%)	571 (0.2%)
	95% CI	*[30.6%-31.0%]*	*[68.6%-69.0%]*	*[1.3%-1.4%]*	*[0.2%-0.3%]*
4th wave		49,216 (40.8%)	71,107 (59.0%)	570 (0.5%)	52 (0.0%)
	95% CI	*[40.5%-41.1%]*	*[58.7%-59.3%]*	*[0.4%-0.5%]*	*[0.0%-0.1%]*

Note: Due to privacy reasons we cannot report exact numbers when numbers are between 0 and 5. Therefore we have censured the exact numbers for deaths in the 1st and 3rd wave for the various age groups. The table still includes the percentages and 95% confidence intervals.
