# Supplementary material for: Pandemic trends in health care use: From the hospital bed to self-care with COVID-19
Source: PLoS One. 2022 Mar 23;17(3):e0265812. doi: 10.1371/journal.pone.0265812 (PMC8942224; doi:10.1371/journal.pone.0265812)
Supplement: Attachment — Submitted filename: Point-by-point_plos_one_new.docx [file pone.0265812.s008.docx]

| We would like to thank the expert reviewers for valuable input, which has helped to improve the quality of our manuscript. Please find below a point-to-point response to your comments and a list of the changes we made in the revised manuscript. | | |
| --- | --- | --- |
| **Comments of Academic Editor** | **Our response** | **Action** |
| Please remove the figure 1 from the introduction and integrate it within a paragraph entitled "study setting" in the methods, where you can describe in a sentence or two the SARS-CoV-2 context in Norway. | We agree. | We removed Fig 1 from the introduction and placed it within a subsection entitled “Study setting” within the Methods section.  Instead of adding one to two sentences on the SARS-CoV-2 context in Norway, we moved the paragraph describing the different waves from the subsection “Statistical analyses” to this new section, as we felt it was more logical to place the paragraph in the new subsection.  We also noticed that we had made an error on line 92. We do not only include hospitalizations with ICD-10 code U071 (confirmed COVID-19), but we also include U072 (suspected COVID-19). This is because these patients have already tested positive for SARS-CoV-2, and in the beginning of the pandemic, hospitals were not coherent in in the use of U071 or U072. This is now corrected in the uploaded manuscript. |
|  |  |  |
